# Hanbury Brown-Twiss effect without two-photon interference in photon counting regime

**DOI:** 10.1038/s41598-017-02408-6

**Published:** 2017-05-19

**Authors:** Bin Bai, Yu Zhou, Ruifeng Liu, Huaibin Zheng, Yunlong Wang, Fuli Li, Zhuo Xu

**Affiliations:** 1 Electronic Materials Research Laboratory, Key Laboratory of the Ministry of Education & International Center for Dielectric Research, Xi’an Jiaotong University, Xi’an, 710049 China; 2 MOE Key Laboratory for Nonequilibrium Synthesis and Modulation of Condensed Matter and Department of Applied Physics, Xi’an Jiaotong University, Xi’an, 710049 China

## Abstract

From quantum point of view, Hanbury Brown-Twiss effect is a result of constructive-destructive two-photon interference. There should be no Hanbury Brown-Twiss effect if there was no two-photon interference. In this paper, we observed Hanbury Brown- Twiss effect in a specially designed experiment, in which two-photon interference is impossible by keeping only one two-photon probability amplitude in the experimental scheme. However, our experimental results can still be interpreted by Glauber’s quantum optical coherence theory. The researches in our paper are helpful to understand the physics of the second-order coherence of light, especially the physics of Hanbury Brown-Twiss effect.

## Introduction

Hanbury Brown-Twiss (HBT) effect, which is also known as two-photon bunching of thermal light, was first observed by Hanbury Brown and Twiss in 1956^1, 2^. At first, HBT interferometer was developed to measure the size of stars by studying the correlation of intensity fluctuations^2^. This method based on HBT interferometer would bring the improvement beyond the Michelson interferometer in measuring the size of stars. In their experiments, two photo-detectors are placed in the far field zone of a chaotic radiation source. The correlation between the signals from the two detectors is measured. They found that independently emitted photons by thermal source were not really independent. Photons in thermal light have the tendency to come in bunches rather than randomly. To understand the results of observations, both quantum and classical theories were employed to interpret this strange phenomenon^3–7^. In classical theory, HBT effect is interpreted by the intensity fluctuation correlation theory^3^. In quantum theory, HBT effect is usually interpreted by two-photon interference^8^. It is later proved that the quantum and classical theories are equivalent when interpreting HBT effect^5, 9^. In quantum optics, classical light can be treated with a proper Glauber P-representation in density matrix formalism^10^. The HBT effect was originally observed for photons in thermal light. Ever since, extensive researches, such as in condensed matter and particle physics^11, 12^, have been motivated by HBT effect. It has been found that HBT effect can be performed with matter^13^, quantum gases^12^, bosons and fermions^14^, interacting photons^15^ and twisted light^16^. The HBT effect^1, 2^, together with Glauber’s quantum optical coherence theory^5, 7^, are usually thought as the cornerstones of modern quantum optics^17^.

In classical intensity fluctuation correlation theory, the HBT effect is due to the correlation of the intensity fluctuation of the signals from two detectors. When two detectors are placed in the same coherence volume of the thermal light field, the correlation between two detected signals can be found^1, 2^. When two detectors are placed in different coherence volumes, there are no correlated intensity fluctuations for two detected signals and no correlation is detected.

In quantum two-photon interference theory, HBT effect is due to the coherent superposition of different but indistinguishable two-photon probability amplitudes^5, 8, 18–21^. There are two different ways for two photons 1 and 2 to trigger a joint detection event at detectors A and B. One way is that photon 1 goes to detector A and photon 2 goes to detector B. In terms of quantum mechanics, this is the two-photon probability amplitudes A_*I*_. The other way is that photon 2 goes to detector A and photon 1 goes to detector B. This way is the probability amplitude A_*II*_. When the two different ways are indistinguishable, the probability of the joint photons detection is *P*_*cc*_ = |*A*_*I*_ + *A*_*II*_|^2^. The HBT effect comes from the interference of different but indistinguishable two-photon probability amplitudes^22–24^. If these two ways are distinguishable, even in principle, there will be no two-photon interference. Thus, no HBT effect should be observed^4^.

In most HBT type experiments, both classical intensity fluctuation correlation and quantum two-photon interference theories give the same prediction. In this paper, we report a tactfully designed experiment in which two theories give different predictions. In our experiment, two pseudo-thermal light beams from different sources with the same intensity fluctuations and orthogonal polarizations are employed. According to the classical intensity fluctuation correlation theory, HBT effect can be observed. On the other hand, no HBT effect should be observed in quantum two-photon interference theory. The photons from laser 1 only go to detector A and photons from laser 2 only go to detector B. In such case, two-photon probability amplitudes interference is impossible because there is only one path left. In our experiments, both spatial and temporal HBT effect are observed. With this research, we find a novel HBT effect without quantum two-photon interference, which shed light on the physics of HBT effect.

## Results

### Theoretical Analysis

HBT is interpreted as the result of the correlation of intensity fluctuations in classical theory. It measures the correlation between the output of two photo-detectors located in two different space-time points (**r**_*A*_, *t*_*A*_) and (**r**_*B*_, *t*_*B*_). The quantity is the second-order coherence function^25^,

1 $${G}^{\mathrm{(2)}}({{\bf{r}}}_{A},{t}_{A};{{\bf{r}}}_{B},{t}_{B})=\langle {{\bf{E}}}_{B}^{\ast }({{\bf{r}}}_{B},{t}_{B}){{\bf{E}}}_{A}^{\ast }({{\bf{r}}}_{A},{t}_{A}){{\bf{E}}}_{A}({{\bf{r}}}_{A},{t}_{A}){{\bf{E}}}_{B}({{\bf{r}}}_{B},{t}_{B})\rangle ,$$

where *I*_*i*_(**r**_*i*_, *t*_*i*_) and **E**_*i*_(**r**_*i*_, *t*_*i*_), *i* = (*A*, *B*), are intensities and electric fields at detector A and B, respectively. For a chaotic radiation, the radiation is the sum of the contribution of many microscopic sources. **E**_*A*_(**r**_*A*_, *t*_*A*_) and **E**_*B*_(**r**_*B*_, *t*_*B*_) as a discrete sum of N components,

2 $$\begin{array}{rcl}{{\bf{E}}}_{A}({{\bf{r}}}_{A},{t}_{A}) & = & \sum _{{\rm{j}}}^{N}{{\bf{E}}}_{A{\rm{j}}}({{\bf{r}}}_{A{\rm{j}}},{t}_{A{\rm{j}}}),\\ {{\bf{E}}}_{B}({{\bf{r}}}_{B},{t}_{B}) & = & \sum _{{\rm{j}}}^{N}{{\bf{E}}}_{B{\rm{j}}}({{\bf{r}}}_{B{\rm{j}}},{t}_{B{\rm{j}}}),\end{array}$$

where *Aj* indicates that the radiation arrives at the detector A from the *j*-th element of the source. The phases of the electric field from each microscopic source is independent and random. Some terms will vanish when the ensemble average is calculated. The function can be simplified as follows^25^,

3 $$\begin{array}{rcl}{G}^{\mathrm{(2)}}({{\bf{r}}}_{A},{t}_{A};{{\bf{r}}}_{B},{t}_{B}) & = & \langle {I}_{A}({{\bf{r}}}_{A},{t}_{A})\rangle \langle {I}_{B}({{\bf{r}}}_{B},{t}_{B})\rangle +{|{{\rm{\Gamma }}}_{AB}^{\mathrm{(1)}}({{\bf{r}}}_{A},{t}_{A};{{\bf{r}}}_{B},{t}_{B})|}^{2}\\  & = & \langle {I}_{A}\rangle \langle {I}_{B}\rangle [1+{|\gamma ({{\bf{r}}}_{A},{t}_{A};{{\bf{r}}}_{B},{t}_{B})|}^{2}],\end{array}$$

where 〈*I*_*A*_〉 and 〈*I*_*B*_〉 are the average intensities recorded by A detector and B detector, ${{\rm{\Gamma }}}_{AB}^{\mathrm{(1)}}$ is the mutual coherence function and *γ* is the first degree of coherence.

The concept of intensity fluctuations is defined as Δ*I*_i_ = *I*_i_ − 〈*I*_i_〉. The correlation between intensity fluctuations is mathematically expressed,

4 $$\langle {\rm{\Delta }}{I}_{A}{\rm{\Delta }}{I}_{B}\rangle =\langle ({I}_{A}-\langle {I}_{A}\rangle )({I}_{B}-\langle {I}_{B}\rangle )\rangle =\langle {I}_{A}{I}_{B}\rangle -\langle {I}_{A}\rangle \langle {I}_{B}\rangle ,$$

Comparing the Eq. (3) and Eq. (4), it is realized that HBT effect is due to the correlation of the intensity fluctuations of the radiation at two detectors. When the intensity fluctuations recorded at two photo-detectors are same, the peak appears and the HBT effect can be observed in the classical interpretation.

From another point of view, the interpretation of two-photon interference is mainly the interference of indistinguishable two-photon probability amplitudes. For the second-order phenomena, the measured quantity is the probability of jointly producing two photo-electron events at space time points (**r**_*A*_, *t*_*A*_) and (**r**_*B*_, *t*_*B*_). For the chaotic light, the density matrix can be written in the following way^5^:

5 $$\hat{\rho }=\sum _{i,j}{P}_{i,j}|{{\rm{\Psi }}}_{i,j}\rangle \langle {{\rm{\Psi }}}_{i,j}|,$$

where P_*i*,*j*_ is the probability of find the radiation in the state |Ψ_*i*,*j*_〉. The state |Ψ_*i*,*j*_〉 can be written explicitly as^26, 27^

6 $$|{{\rm{\Psi }}}_{{\rm{i}},{\rm{j}}}\rangle =|{{\rm{\Psi }}}_{{\rm{i}}}\rangle |{{\rm{\Psi }}}_{{\rm{j}}}\rangle =A\int {\rm{d}}\omega {\rm{f}}(\omega ){{\rm{e}}}^{-{\rm{i}}\omega {t}_{0i}}{a}^{\dagger }(\omega )|0\rangle \int {\rm{d}}\omega ^{\prime} {\rm{f}}(\omega ^{\prime} ){{\rm{e}}}^{-{\rm{i}}\omega ^{\prime} {t}_{0j}}{a}^{\dagger }(\omega ^{\prime} )|0\rangle $$

where t_0*i*_ represents the creation time of every independent wave packet. The second-order Glauber correlation function is^5, 7^

7 $${G}^{\mathrm{(2)}}({{\bf{r}}}_{A},{t}_{A};{{\bf{r}}}_{B},{t}_{B})=Tr[\hat{\rho }{{\bf{E}}}_{A}^{(-)}({{\bf{r}}}_{A},{t}_{A}){{\bf{E}}}_{B}^{(-)}({{\bf{r}}}_{B},{t}_{B}){{\bf{E}}}_{B}^{(+)}({{\bf{r}}}_{B},{t}_{B}){{\bf{E}}}_{A}^{(+)}({{\bf{r}}}_{A},{t}_{A})].$$

When the density matrix in chaotic light is employed, the second-order correlation function should be written as^26, 28^

8 $${G}^{\mathrm{(2)}}({{\bf{r}}}_{A},{t}_{A};{{\bf{r}}}_{B},{t}_{B})=\sum _{i,j}\langle {{\rm{\Psi }}}_{i,j}|{{\bf{E}}}_{A}^{(-)}({{\bf{r}}}_{A},{t}_{A}){{\bf{E}}}_{B}^{(-)}({{\bf{r}}}_{B},{t}_{B}){{\bf{E}}}_{B}^{(+)}({{\bf{r}}}_{B},{t}_{B}){{\bf{E}}}_{A}^{(+)}({{\bf{r}}}_{A},{t}_{A})|{{\rm{\Psi }}}_{i,j}\rangle .$$

In the HBT experiment, photons trigger two detectors (D_*A*_ and D_*B*_). In terms of two-photon amplitudes, the function is calculated to be^4, 18, 28^

9 $$\begin{array}{rcl}{G}^{\mathrm{(2)}}({{\bf{r}}}_{A},{t}_{A};{{\bf{r}}}_{B},{t}_{B}) & = & \sum _{i,j}{|{{\bf{A}}}_{i\to A;j\to B}({{\bf{r}}}_{A},{t}_{A};{{\bf{r}}}_{B},{t}_{B})+{{\bf{A}}}_{i\to B;j\to A}({{\bf{r}}}_{A},{t}_{A};{{\bf{r}}}_{B},{t}_{B})|}^{2}\\  & = & \sum _{i,j}{|{{\bf{A}}}_{i\to A;j\to B}|}^{2}+\sum _{i,j}{|{{\bf{A}}}_{i\to B;j\to A}|}^{2}+2Re\sum _{i,j}{{\bf{A}}}_{i\to A;j\to B}^{\ast }{{\bf{A}}}_{i\to B;j\to A},\end{array}$$

where two quantities **A**_*i*→*A*;*j*→*B*_ and **A**_*i*→*B*;*j*→*A*_ are two-photon amplitudes. **A**_*i*→*A*;*j*→*B*_ expresses that photon *i* is recorded by detector *D*_*A*_ and photon *j* is recorded by detector *D*_*B*_. At the same time, **A**_*i*→*B*;*j*→*A*_ expresses that photon *i* is recorded by detector *D*_*B*_ and photon *j* is recorded by detector *D*_*A*_.

The law of combing amplitudes is used in the joint photo-detection event. In quantum two-photon interference theory, the superposition of probability amplitudes takes place due to two alternative, different and indistinguishable paths. It shows that the HBT effect can be observed due to the superposition of amplitudes when the paths are indistinguishable.

### Experimental verification

**Figure 1:**
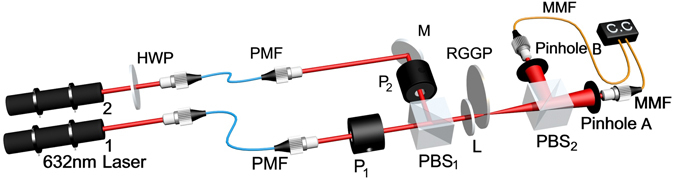
Experimental setup for measuring HBT effect without two-photon interference. Laser 1 and 2 are single-mode continuous-wave He-Ne lasers. HWP is a half wave plate. PWF is the single mode polarization-maintaining fiber. *P*_1_ and *P*_2_ are two polarizers, which are employed to keep the polarizations of two beams orthogonal. PBS_1_ and PBS_2_ are two polarization beam splitters. Two beams are combined into one beam at PBS_1_ and the combined beam is focused by the lens (L) on the rotating ground glass plate (RGGP). PBS_2_ splits the combined beam into two beams of light. MMF is the multi-mode fiber, which are employed to collect photons and transfer light to two single-photon detectors. It is measured by a HBT intensity interferometer.

The experimental setup is shown in Fig. 1. In the experiment, two He-Ne lasers with wavelength at 632.8 nm are used. The polarizations of both lasers are horizontal initially. A half wave plate (HWP) behind laser 2 changes the horizontal polarization of the light beam from laser 2 into vertical polarization. Two beams pass through two single mode polarization-maintaining fibers and two polarizers P_1_ and P_2_. P_1_ is set to horizontal polarization and P_2_ is set to vertical polarization respectively to keep the polarizations of two beams unchanged. We then proceed our experiments in three major steps.

**Figure 2:**
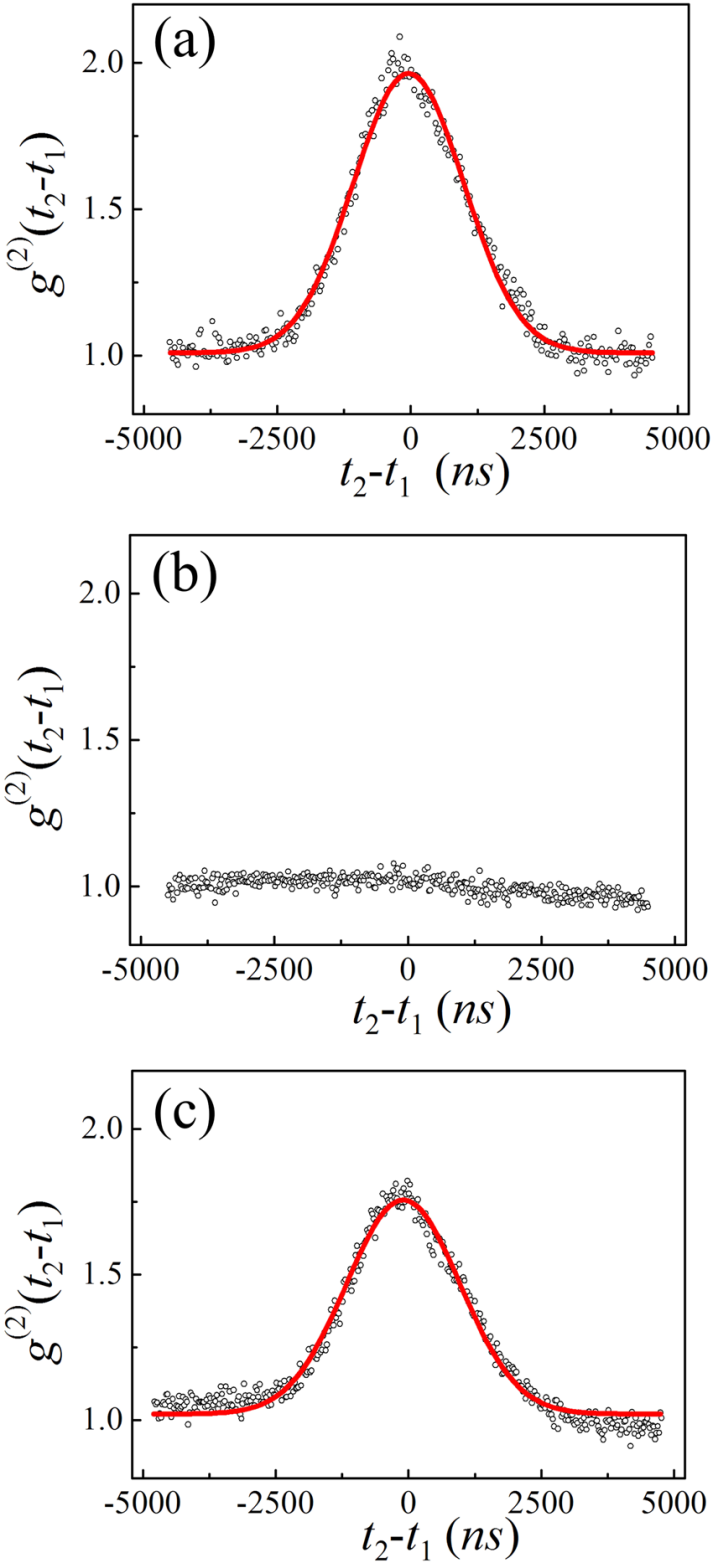
The results of temporal HBT effect. (**a**) Is the measured result in Step I. The visibility of the peak is about 31.5%. (**b**) Is the measured result in Step II. No HBT effect can be observed. (**c**) Is the measured result in Step III. The HBT effect is observed and its FWHM is 2520 ns. The visibility of the peak is 26.3%.

In Step I, the HBT intensity interferometer is tested with pseudo-thermal light. Laser 1 is on and laser 2 is off. The beam is focused by a lens (L) onto a rotating round ground glass plate (RGGP) to generate pseudo-thermal light^29^. Because the generated pseudo-thermal light is also horizontal polarized, the light passes through the polarized beam splitter 2 (PBS_2_) can only reach single photon detector A (which detects the photons through Pinhole A). The coincidence count in this measurement is almost zero because there is almost no light reaching single photon detector B (which detects the photons through Pinhole B). To test the HBT intensity interferometer, an additional half wave plate is placed between the first polarized beam splitter (PBS_1_) and the rotating ground glass (not shown in Fig. 1). The half wave plate rotates the horizontal polarized laser beam from laser 1 into 45° with respect to the horizontal direction. The light beam is split into two after it passes through PBS_2_. Two detectors in the HBT intensity interferometer are triggered by photons. The result is shown in the Fig. 2(a). The full-width-of-half-maximum (FWHM) of the correlation peak in time domain is about 2437 ns, which is determined by the rotating speed of the ground glass^29^. The measured result shows that the HBT intensity interferometer works properly. The same test is repeated by turning laser 2 on and keeping laser 1 off. The measured result is similar as the previous one.

In Step II, laser 1 and laser 2 are both turned on. The polarization of the light beam from laser 1 is horizontal and the polarization from laser 2 is vertical. Two beams are combined into one beam at PBS_1_. The combined beam is focused by the lens (L) onto RGGP. The areas of two beams on the RGGP are different. Two sets of different speckles can be obtained. The first set of speckles is from laser 1 with horizontal polarization and can only reach detector A. The second set of speckles is from laser 2 with vertical polarization and can only reach detector B. The intensity fluctuations of two sets of speckles are different because they are generated by laser light scattered from different areas on the RGGP. The scattered light is split by PBS_2_ into two beams due to their mutual-orthogonal polarizations. Detector A only receives photons from laser 1 with horizontal polarization and detector B only receives photons from laser 2 with vertical polarization. The measured result is shown in Fig. 2(b). The *g* ^(2)^(*t*_2_ − *t*_1_) function is flat and no HBT effect is observed. Both classical intensity fluctuation correlation and quantum two-photon interference theories can explain the measured results in Step I and II properly.

In Step III, the experimental setup is same as the one in Step II except that two beams are carefully focused *on the same spot* of RGGP. There are two sets of pseudo-thermal light with identical spatial and temporal intensity fluctuation distribution with mutual-orthogonal polarizations. The pseudo-thermal through PBS_2_ is measured by the HBT intensity interferometer. Light from laser 1 passes through two beam splitters and only triggers detector A due to its horizontal polarization. For the same reason, light from laser 2 is reflected by PBS_2_ and only triggers detector B.

**Figure 3:**
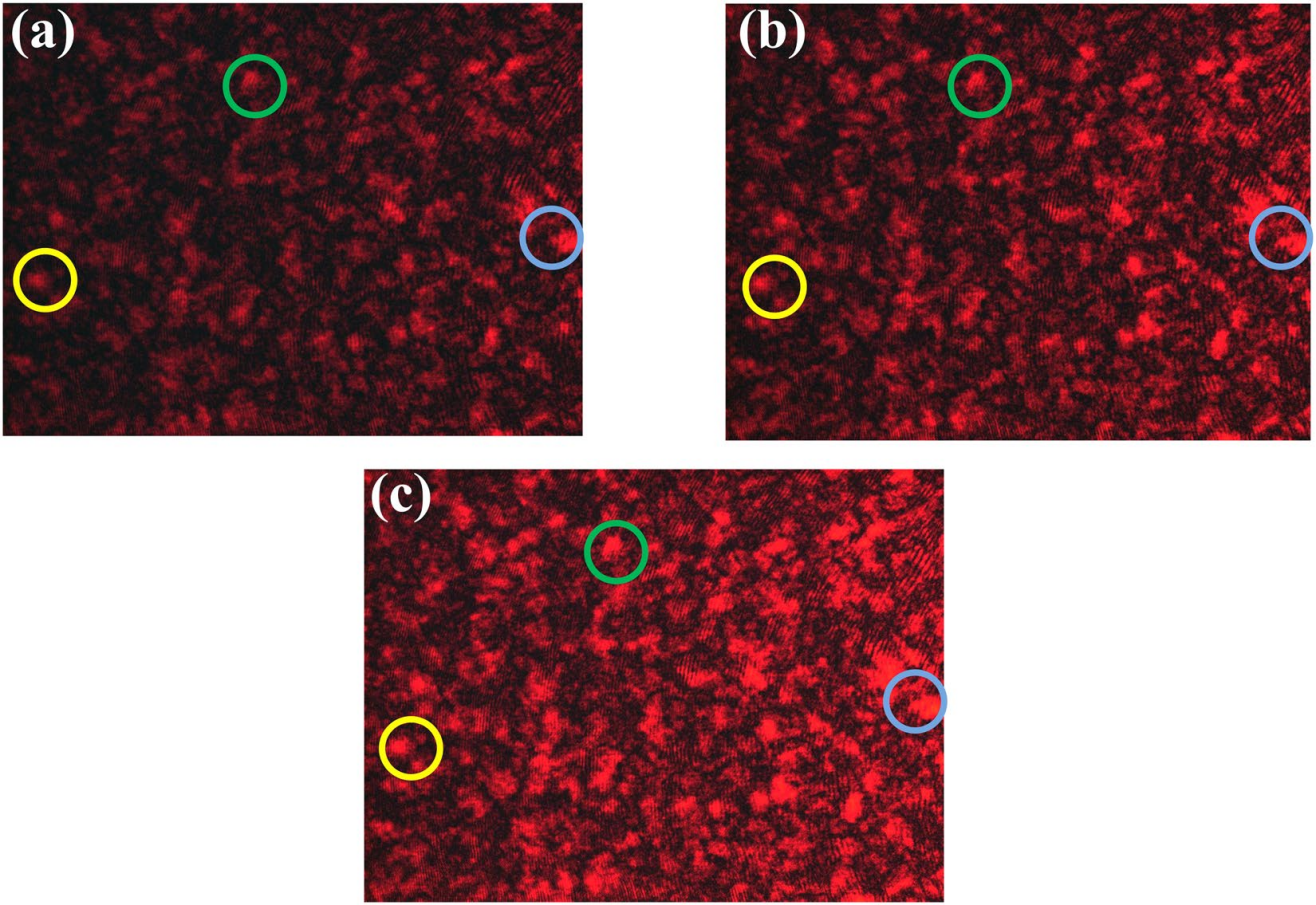
The speckle patterns in Step III. (**a**) Is the speckle pattern of the reflected light with vertical polarization through PBS_1_ from laser 2. (**b**) Is the speckle pattern of the transmitted light with horizontal polarization from laser 1. (**c**) Is the image of the speckle pattern when both laser light beams are focused on the RGGP at the same time. Though the intensities of light in (**a**,**b**) are not identical, the speckle patterns are almost same.

We carefully make the focused horizontal-polarized (from laser 1 only) and vertical-polarized (from laser 2 only) laser beam on the same spot of the ground glass plate in Step III. The speckle patterns are measured by placing a CCD after the RGGP. Figure 3(a) is the speckle pattern of the reflected light with vertical polarization through PBS_1_ from laser 2. Figure 3(b) is the speckle pattern of the transmitted light with horizontal polarization from laser 1. Figure (c) is the image of the speckle pattern when both laser light beams are focused on the RGGP at the same time. The generated speckle patterns are almost identical, which can be seen by the circles shown in Fig. 3(a–c). Since the two sets of pseudo-thermal light have identical intensity fluctuations when the ground glass is rotating, HBT effect is expected to be observed according to classical intensity fluctuation correlation theory. On the other hand, it is predicted by two-photon interference theory that there will be no HBT effect due to there is only one path left, that is photon 1 goes to detector A and photon 2 goes to detector B.

**Figure 4:**
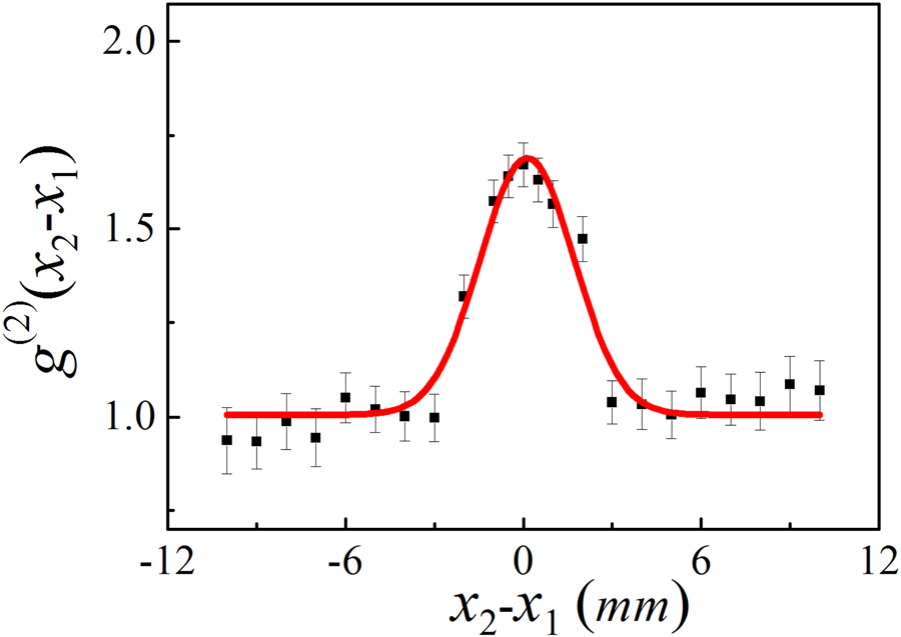
The measured spatial HBT effect in Step III. It is measured by scanning detector A horizontally when detector B is fixed. The FWHM is 3.75 mm and the visibility of the peak is about 25.4%.

The result in Step III is shown in Fig. 2(c). Firstly, the second-order coherence function is measured in temporal domain. A peak is observed, which means that there is temporal correlation between the light field at points of detectors A and B. Then the second-order spatial coherence function is measured. The coincidence counting rate is measured by scanning detector A horizontally when detector B is fixed. The result is shown in Fig. 4. The FWHM is 3.75 mm and the visibility of the peak is about 25.4%. The spatial HBT effect is also observed.

## Discussion

In Step I, when one light beam (from laser 1 or 2) with polarization set to 45° passes through RGGP and PBS_2_, there are two sets of identical speckle patterns. The detectors would record the same intensity fluctuations or photon number fluctuations as long as they are in symmetric positions. Therefore, the observed HBT effect can be interpreted by the classical intensity fluctuation correlation of the light field at those two detectors^5, 25^. On the other hand, one beam of light is split into two by PBS_2_ because the polarization of light is 45° with respect to horizontal polarization. There are two different but indistinguishable paths for the joint photo-detection event. The HBT effect can be interpreted as the result of the two-photon interference^4, 8^.

In Step II, both classical and quantum theories can explain the experimental results, too. In classical theory, the speckles of two beams of light are different so their intensity fluctuations are different. When it is measured by the standard HBT intensity interferometer, there is no peak due to the intensity fluctuations recorded by detector A and B are different. In quantum two-photon interference theory, the photons recorded by two detectors are distinguishable due to mutual-orthogonal polarizations. The photons from laser 1 with horizontal polarization can only trigger detector A and the photons from laser 2 with vertical polarization can only trigger detector B. According to two-photon interference theory, no HBT effect can be observed because there is only one two-photon probability amplitude left in the experiment.

**Figure 5:**
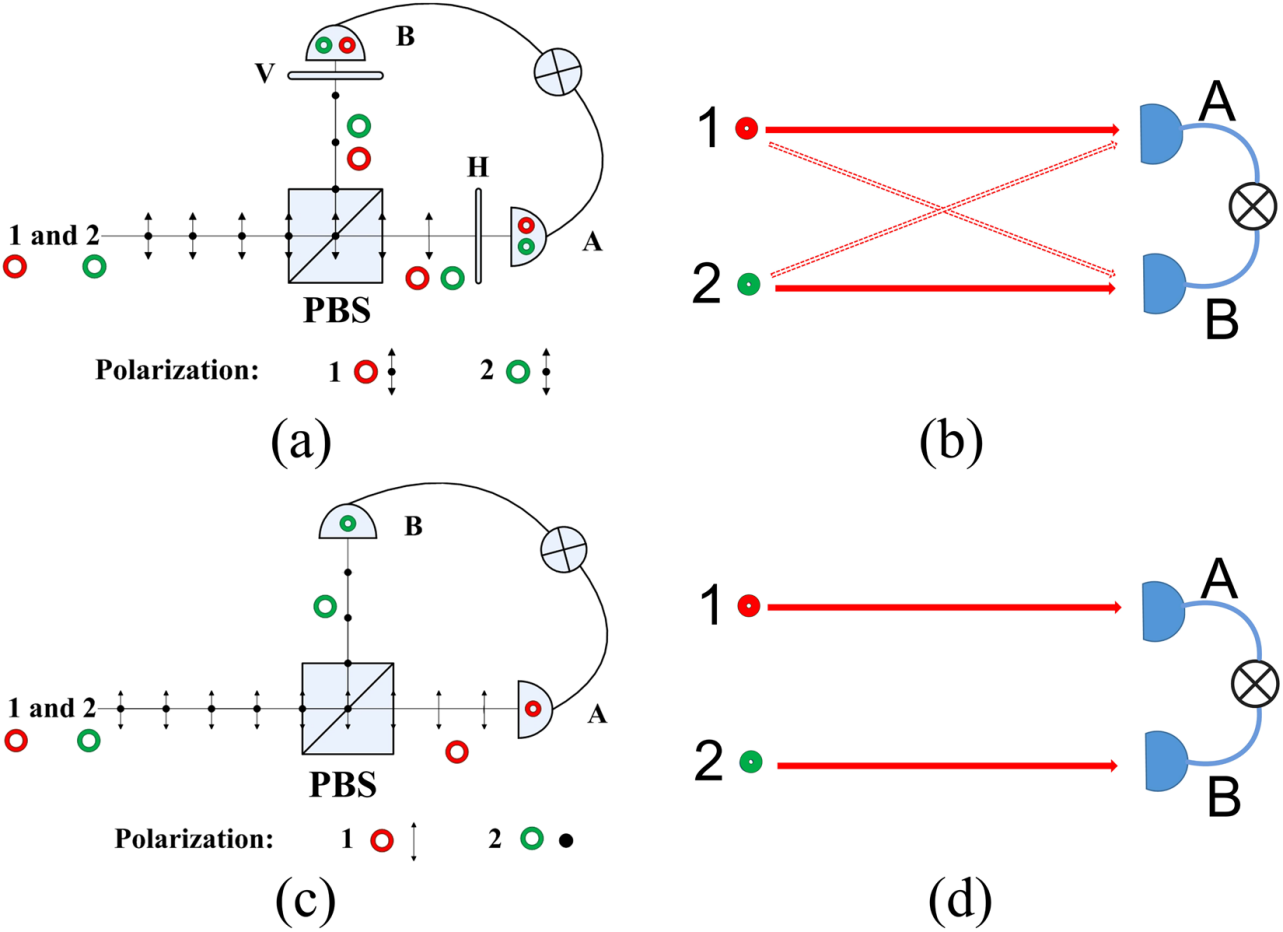
The difference of experiments between the HBT effect for the photons with orthogonal polarizations. (**a**) Is the scheme of experiment to certify the effect of HBT when the photons have the orthogonal polarizations. The polarization of photon 1 and 2 both are 45° with respect to horizontal polarization. (**b**) Shows the paths in which photon 1 and 2 reach the detectors. (**c**) Is the scheme in Step III of our experiment. Photon 1 with horizontal polarization only goes to detector A and photon 2 with vertical polarization only goes to detector B. (**d**) Shows only one path to trigger two-photon coincidence count.

In Step III, classical intensity fluctuations and quantum two-photon interference theory give different predictions on whether HBT effect can be observed or not. From classical point of view, HBT effect can be observed. The horizontal-polarized (from laser 1 only) and vertical-polarized (from laser 2 only) laser beam are focused on the same spot of RGGP. It makes sure that two sets of speckle patterns are identical while their polarizations are orthogonal. After PBS_2_, the vertical-polarized light is reflected to detector B and the horizontal-polarized light is transmitted to detector A. Since two sets of pseudo-thermal light have identical intensity fluctuations when the ground glass is rotating, HBT effect is expected according to intensity fluctuation correlation theory. On the other hand, it is predicted by two-photon interference theory that there is no HBT effect. The photons from laser 1 only go to detector A and photons from laser 2 only go to detector B as shown in Fig. 5(c,d). There is only one path left and there is no two-photon interference.

The experiment in Step III is different from that of Zhai. *et al*.^19^. In the experiment of Zhai. *et al*., the orthogonal polarized photons through the ground glass can reach both detectors D_1_ and D_2_. In their second experiment, they observed HBT effect which is from the photon bunching of two horizontal-polarized photons or two vertical-polarized photons, but never only from one horizontal-polarized photon and one vertical-polarized photon. Our experiment in Step III is different from experiments which certify the HBT effect between the photons with orthogonal polarizations as shown in Fig. 5(a,b). In Fig. 5(a,b), the polarization of photon 1 and 2 both are 45° with respect to horizontal polarization. The photon 1 and 2 are indistinguishable for detectors even though the existence of the polarizers. Though the photons with orthogonal polarizations reach the detectors, there are still two-photon probability amplitudes interference as shown in Fig. 5(b). For the experiment in Fig. 5(a), the classical and quantum theory should give the same prediction on whether HBT effect will be observed or not. However, our experiment is different as shown in Fig. 5(c,d). There is no chance for photons from laser 1 go to detector B nor photons from laser 2 go to detector A. All coincidence events must come from that one photon with horizontal polarization from laser 1 triggers detector A and one photon with vertical polarization from laser 2 triggers detector B. In such case, two-photon interference is impossible because there is only one path left.

In Glauber’s quantum optical coherence theory, the HBT effect is described by the second-order coherence function *g* ^(2)^. It can be expressed in terms of the mean and mean-square photon numbers as^5, 25^

10 $${g}^{\mathrm{(2)}}(\tau )=\frac{\langle n(n-\mathrm{1)}\rangle }{{(\langle n\rangle )}^{2}}=\frac{\langle {n}^{2}\rangle -\langle n\rangle }{{(\langle n\rangle )}^{2}}=1+\frac{{({\rm{\Delta }}n)}^{2}-\langle n\rangle }{{(\langle n\rangle )}^{2}}.$$

The photon number probabilities for different light source would get different value of *g* ^(2)^. Here, the photon number probability is reflected in the density operator. In our experiment, pseudo-thermal light is generated from RGGP. The generated photons follows as the photon number probability of chaotic light^29^. For the chaotic light as the thermal excitation of photons, the density operator can be expressed as^25^

11 $$\hat{\rho }=\sum _{n}\frac{{\langle n\rangle }^{n}}{{(1+\langle n\rangle )}^{1+n}}|n\rangle \langle n|.$$

It is shown that the photon-number variance is related to the mean by

12 $${({\rm{\Delta }}n)}^{2}={\langle n\rangle }^{2}+\langle n\rangle .$$

The chaotic light, whose photon-number variance exceeds 〈*n*〉, is said to exhibit super-Poissonian fluctuations. The bunching effect would occur and the degree of second-order coherence can be 2. The HBT effect can be observed.

In Step II and Step III of our experiment, the pseudo-thermal light can be generated when the light from two lasers is focused on RGGP. Photons with horizontal polarization from laser 1 and photons vertical polarization from laser 2 both follow the number probability of chaotic light. In Step II, when the photons from two lasers are focused on the different spot of RGGP, light from laser 1 and 2 have the different intensity fluctuation. Detector A would only get photons from laser 1 and detector B would only get photons from laser 2 due to their polarization. In such case, although photons triggering detector A and B both follow the number probability of chaotic light, their photon-number fluctuations are independent of each other at any point in time. When two-photon coincidence count between detector A and B is measured, no HBT effect can be observed. In Step III, when the photons from two lasers are focused on the same spot of RGGP, light from laser 1 and 2 have the same intensity fluctuation. In such case, not only photons triggering detector A and B both follow the number probability of chaotic light, but also the same photon-number fluctuation can be measured. The photons detected by two detectors both follow super-Poissonian fluctuations and their photon-number fluctuation are same. When two-photon coincidence count is measured, HBT effect can be observed.

## Conclusion

In intensity fluctuation correlation and two-photon interference theories, the HBT effect can usually be understood properly. The classical intensity fluctuation correlation theory emphasizes that the HBT effect is attributed to the correlation between the same intensity fluctuations. While the quantum two-photon interference theory emphasizes that the HBT effect is attributed to two-photon interference between indistinguishable probability amplitudes. In this paper, we report an experiment in which the classical theory and two-photon interference theory give different predictions. Classical theory predicts HBT effect can be observed. Two-photon interference theory predicts HBT effect can not be observed. The experimental results show that both the temporal and spatial HBT effects are observed. It does not mean quantum theory can not interpret the experimental results. It only means that there is no two-photon interference in the observed HBT effect in our experiment.

We noticed that in recent research the correlation of HBT effect in ghost imaging is separated into two parts–the classical part and the quantum part on the criteria of quantum discord^30^. According to the paper mentioned, the quantum correlation and classical correlation does exist in any intensity of light. When the light is very weak, the quantum part is bigger than classical part. With the increase of light intensity, the quantity of classical correlation will exceed that of quantum correlation. In our experiment, the correlation of HBT is recognized as classical intensity fluctuation correlation because the quantum two-photon interference interpretation is ruled out by our setup. And the experimental results can be interpreted by Glauber’s quantum theory.
